# Fatigue in Older Adults Postmyocardial Infarction

**DOI:** 10.3389/fpubh.2016.00055

**Published:** 2016-04-11

**Authors:** Patricia Barton Crane, Jimmy T. Efird, Willie Mae Abel

**Affiliations:** ^1^College of Nursing, East Carolina University, Greenville, NC, USA; ^2^Department of Nursing, University of North Carolina at Charlotte, Charlotte, NC, USA

**Keywords:** fatigue, aging, myocardial infarction

## Abstract

**Objective:**

The purpose of this study was to comprehensively examine putative factors that may independently contribute to fatigue and subsequent persistence of fatigue in elderly adults 6–8 months post-myocardial infarction (MI). Studies suggest cardiac function, comorbidities, daytime sleepiness, depression, anemia, interleukins, and social support are correlates of fatigue; however, no studies have systematically examined these factors 6 months post-MI in an aging population.

**Methods:**

Study participants included 49 women and men (*N* = 98) ages 65–91 who were 6–8 months post-MI. Data collection included the demographic health status questionnaire (heart rate, blood pressure, body mass index, and medications), fatigue-related comorbidity scale, revised Piper fatigue scale, Epworth sleepiness scale, geriatric depression scale, social provisions scale, and venous blood tests (B-natriuretic peptide, hemoglobin, and interleukin-6).

**Results:**

Fatigue persisted after MI in 76% of older men and women with no difference by sex. Only depression scores (*P*_trend_ = 0.0004) and mean arterial pressure (*P*_trend_ = 0.015) were found to be linearly independent predictors for fatigue, controlling for age, Il-6 levels, and body mass index.

**Conclusion:**

Post-MI depression and mean arterial blood pressure are important to assess when examining fatigue post-MI in older populations.

## Introduction

The majority of annual myocardial infarctions (MIs) occur in those aged 65 and older with approximately 29% being recurrent events (1). These high recurrence rates may be related to a lack of secondary prevention behaviors. One of the most effective secondary prevention behaviors after an MI is physical activity. However, fatigue is one of the most frequently reported barriers to participation in physical activity (2). Fatigue reported with an MI is greater than fatigue associated with arthritis, diabetes, lung disease, migraines, or hypertension (3) and is the most common symptom reported 5 months after MI in both men and women (4). This is concerning because fatigue is associated with poorer cardiac outcomes, including increased cardiac readmissions and sudden cardiac death (5).

Fatigue, tiredness, and exhaustion are commonly used descriptors of loss of stamina related to physical, emotional, and cognitive activity and are similar to mood descriptors such as “sad” and “blue.” Just as when “sadness” becomes a consistent emotion that contributes to a clinical diagnosis of depression, fatigue that persists is a significant problem affecting behavior and quality of life. Typically fatigue is viewed as a multidimensional symptom consisting of a physical/behavioral, perceptual (sensory or affect), and cognitive components (6).

Research on fatigue in a post-MI population has largely been limited to the near term occurrence (3–4 months) following the MI (7). However, fatigue assessed in this short period may be associated with physiological recovery (8) and thus may not be the same as persistent fatigue that affects participation in physical activity and other behaviors associated with secondary prevention. Furthermore, studies on fatigue post-MI have included either small numbers of elderly adults or no elderly adults; therefore, we cannot translate the findings to elderly adults who have the greatest incidence of MI and are at the highest risk for recurrent MI.

Measuring fatigue at least 6 months after MI is important to allow for (1) physiological recovery and myocardial tissue to repair, (2) completion of cardiac rehabilitation Phase II, (3) stabilization of a medication regimen, (4) stabilization of emotional distress and functional ability (9), and (5) accurate capture of fatigue and factors associated with fatigue that persists over time post-MI. While previous studies with MI and other clinical populations suggest that cardiac function (10), comorbidities (11), sleep (12), depression (12), anemia (13), interleukin 6 (IL-6) (11), and social support (14) are independently associated with fatigue, no studies have systematically examined these factors and fatigue post-MI. The study of these comorbidities is especially important for the elderly population who has the highest incidence of MI. Therefore, the purpose of this study was to comprehensively examine putative factors that may independently contribute to fatigue and subsequent persistence of fatigue in elderly adults 6–8 months post-MI.

We hypothesized that older age, female sex, more comorbidities, β-blocker usage, lower heart rate, poorer cardiac function (as measured by higher B-natriuretic peptide, BNP), anemia, higher IL-6, greater daytime sleepiness, more depression, and lower social support would explain fatigue 6–8 months post-MI among elderly adults.

## Materials and Methods

### Setting and Participants

The setting was a large metropolitan health-care system serving patients in the mid-central region of North Carolina. Adults aged 65 and older who had been discharged with a diagnosis of acute MI [International Classification of Diseases (ICD)-9 codes 410.1–410.9] were recruited for the study. Inclusion criteria were the verbal validation of an MI by each participant and the ability to speak English. Exclusion criteria were used to control for issues that might affect the independent variables or symptom recall and included the following: a memory deficit (recall), as identified by a score of 6 or above on the Abbreviated Mental Test (15), taking antidepressant medication for depression (depression), major surgery within the last 6 months (inflammation), or chemotherapy for cancer within the last 12 months (inflammation). The study was approved by the university and medical center’s institutional review boards, and informed consent was obtained from each participant.

### Design and Procedure

We used a cross-sectional descriptive design to identify putative factors that independently contributed to fatigue and subsequent persistence of fatigue in the months following the occurrence of an MI. Potential participants who agreed to release their name and telephone number to a site collaborator from the medical center were contacted by telephone to further explain the study, answer any questions, confirm eligibility, and establish a time to collect data. Participants were met at their chosen location, and data were collected. The procedure for data collection included (a) completing the demographic health status (DHS) questionnaire, a form to gather data related to family history of heart disease, risk status, cardiac rehabilitation attendance, and other queries, including obtaining height and weight using a standardized scale, measuring the pulse and blood pressure, querying the participant about comorbidities using the fatigue-related comorbidity scale, visually observing and validating with the participant that they were taking the medication as directed for the specified comorbidity, and documenting all medications and dosages on the DHS questionnaire; (b) collecting a venous blood sample to measure BNP, hemoglobin (Hgb), and IL-6; and (c) completing the revised Piper fatigue scale (RPFS), the Epworth sleepiness scale (ESS), the geriatric depression scale (GDS), and the social provisions scale (SPS).

### Measures

Demographic data were collected with a standard form. Additionally, time of data collection was recorded to examine the relationship of time of day to results. Comorbidities were measured by the fatigue-related comorbidity scale. This instrument was developed by the first author by adapting and combining the chronic disease score and the Charlson comorbidity index to obtain a comprehensive list of comorbidities. “The validity of a comorbidity index is assessed by how well the index predicts those outcomes …” (p. 892) (16). Comorbidity scores could range from 0 to 67 with higher scores associated with higher comorbidities.

A sample of venous blood (10 cc of whole blood) was obtained using a 23-gauge needle and placed in standardized blood vials. One vial was centrifuged using a portable centrifuge, and the serum was extracted using a pipette and placed in the appropriate vial designated by the lab’s protocol.

Ventricular function was measured by the BNP level. BNP is more sensitive (90%) than clinical judgment (49%) in diagnosing heart failure, with greater sensitivity than atrial natriuretic peptides in the elderly (17). BNP levels reflect both systolic and diastolic failure (18), and an increased BNP indicates reduced ventricular systolic and diastolic function. The BNP was measured using a venous sample and the ADVIA Centaur BNP assay. A receiver operator curve analysis indicated that the area under the curve for the ADVIA Centaur BNP assay is 0.919 with a 95% CI of 0.904–0.934 (19).

Anemia was determined by the Hgb lab value and recorded as a dichotomous variable. Using the criteria from the World Health Organization (20), Hgb levels below 12 g/dL in women and below 13 g/dL in men signified the presence of anemia. Hgb was measured using an automated cell counter of EDTA whole blood.

IL-6 was measured using an IMMULITE Analyzer in EDTA plasma. A sample of 100 μL of serum was required, and the sample could be stored for 24 h at 2–8°C. This test has a 99% specificity, an analytical sensitivity of 5 pg/mL, and has reliability estimates of 0.994–0.998 (19).

The RPFS is a 27-item tool that measures four subjective dimensions of fatigue; it is one of the few fatigue tools that has been used with elderly adults (21). Prior to administering the RPFS, the participants were asked if they had experienced fatigue, tiredness, or exhaustion after their MI that differed from fatigue experienced prior to their MI. This question was to clarify persistent fatigue post-MI from fatigue they would note as “normal” or “typical” fatigue. If they answered no to the question, which is their fatigue was the same or less than fatigue experienced prior to MI, they then received a 0 score on the RPFS. If they answered yes, they proceeded to complete the RPFS, and this fatigue was noted as “atypical” fatigue. The 22 items of the RPFS are on a 0–10 scale. The total fatigue score is computed by summing all 22 items and can range from 0 to 220. Reliability and validity estimates are moderate to strong (22), and internal consistency reliability estimates are above 0.80 (6). In this study, the standardized alpha for the total instrument was 0.95.

Daytime sleepiness was measured using the ESS (23). This scale is composed of eight scenarios for which respondents rate the likelihood of falling asleep, from 0 = never doze to 3 = high chance of dozing, for a total possible score of 24. Higher scores indicate more daytime sleepiness. ESS scores are significantly associated (*P* < 0.01) with the multiple sleep latency test at night and with polysomnography (23). Test–retest reliability is reported at 0.82 (24), and Cronbach’s alpha was 0.78.

Depression was measured using the 15-item GDS (25). This scale was selected over the 5-item scale to better approximate a normal distribution and satisfy assumptions for statistical analyses (25). The 15-item scale contains statements to which respondents answer either “yes” or “no.” Five items are reversed; the “no” receives a point, and these items are added to the “yes” items of the other statements, with possible scores ranging from 0 to 15. A cutoff-point score of 5–6 produced 85% sensitivity and 74% specificity in screening for depression (26). Internal consistency of 0.83 was found in a study of elderly adults (27). Cronbach’s alpha for this study was 0.81.

Perceived social support was measured using the SPS, a 24-item questionnaire developed (28) to assess the six provisions of social relationships described by Weiss: guidance (advice or information), reliable alliance (assurance that others can be counted on in times of stress), reassurance of worth (recognition of one’s competence), attachment (emotional closeness), social integration (a sense of belonging to a group of friends), and opportunity for nurturance (providing assistance to others). Each provision is measured by four items: two that describe the presence and two that describe the absence of the provision. The instrument uses a 4-point Likert-type scale (1 = strongly disagree and 4 = strongly agree) and contains both positive and negative scoring items to help control response patterns. Internal consistency ranges from 0.85 to 0.92 (29). The coefficient alpha for this study was 0.90.

All forms and instruments were printed in 14-point font on white paper to ensure ease of reading. Participants were provided the opportunity to read or have the information read to them. The average time to complete data collection was 90 min, and participants were offered breaks throughout. Each participant received a $20 gift certificate upon completion of data collection.

### Statistical Analyses

Categorical variables were expressed as frequency and percentage, whereas continuous variables were reported as median (Md) and interquartile range (IQR). We also plotted the military time of the interview against the overall fatigue score (the overall score for each subscale) to identify the magnitude of fatigue in relation to the timing of the interview. Because no pattern was observed, the timing of the interview was not included as a covariate in the analysis.

Fatigue was considered as an overall score. To analyze the outcome response of fatigue, we categorized the sample into those reporting post-MI fatigue that differed from fatigue prior to their MI and those reporting no difference in fatigue post-MI.

A Fisher’s exact test was used to determine if the proportion of women with fatigue was greater than the proportion of men with fatigue. The Deuchler–Wilcoxon exact procedure for continuous variables was used to compare the Mds of men and women on the overall fatigue scale. In addition, we used graphical assessments to visually inspect significant differences in the distributions of fatigue scores for men and women.

A Poisson regression model was used to compute adjusted relative risks (RR) for fatigue. This analysis determines the contribution each independent variable makes alone and in combination with other independent variables in predicting the outcome response, the presence of fatigue. Model fit was assessed using deviance-based diagnostic plots. To control the influence of outliers, each continuous predictor variable was quartiled from low to high values, with the first quartile serving as the reference point (1.0). Each of the following three quartiles was then compared to the referent. For example, if quartile 3 had an RR of 1.6, those in this quartile were 60% more likely to have the variable than the referent. Tests for trend, to denote an increased or decreased risk associated with a linear change in the predictor variables, are presented where appropriate, and *P*-values were computed using a likelihood ratio procedure. We computed 95% confidence intervals for model parameters using robust variance “sandwich” estimates. Variables included in the multivariable model were those found to be statistically significant in univariable analyses (e.g., confidence intervals did not include unity in at least one category) or displayed a significant linear trend. The pairwise addition of other variables did not substantively change the results. *P*-values <0.05 were considered statistically significant. Results were not adjusted for multiplicity in this pilot analysis. SAS Version 9.3 (Cary, NC, USA) was used for all analyses.

## Results

A sample of 49 women and 49 men (*N* = 98) ranging in age from 65 to 91 (Md = 76, IQR = 10) and 6–8 months post-MI participated in this study. Most were white (84%), married (51%), had a high school education or less (61%), and reported an average household income less than $40,000 (57%). The majority (72%) of participants reported 4–7 comorbidities. The Md comorbidity score for the total sample was 11 (IQR = 5). The Md systolic blood pressure for those who indicated fatigue was 120 (IQR = 22) and for not fatigued was 135 (IQR = 41). The Md diastolic blood pressure for those indicating fatigue was 64 (IQR = 12) and for those with no fatigue was 70 (IQR = 13). See Table 1 for descriptions of the demographics and predictor variables by fatigue presence.

**Table 1:** **Sample characteristics according to the presence of fatigue after myocardial infarction**.

Characteristic	Fatigue (*N* = 74) *n* (%) Median [IQR]	No fatigue (*N* = 24) *n* (%) Median [IQR]	*P*-value^a^
**Demographics**			
Age (years)	77 [11]	73 (7)	0.046
Female	40 (54)	9 (38)	0.24
High school or greater	50 (68)	19 (79)	0.32
Income ≥ $20,000	44 (59)	18 (75)	0.23
Married	37 (50)	13 (54)	0.82
White	61 (82)	21 (88)	0.75
**Medical history/laboratory measurements**			
B-natriuretic peptide (pg/mL)	118 [152]	118 [235]	1.0
β-blocker use	64 (86)	20 (83)	0.74
Body mass index (kg/m^2^)	28 [8]	29 [6]	0.29
Heart rate (beats/min)	64 [13]	63 [13]	0.85
Hemoglobin (g/dL)	13 [2]	13 [1]	0.21
Interleukin-6 (μg/dL)	5 [3]	4 [4]	0.034
Mean arterial pressure (mmHg)^b^	82 [17]	92 [16]	0.0062
**Measurement scales/scores**			
Comorbidities score	10 [5]	10 [5]	0.58
Epworth sleepiness scale	6 [7]	6 [5]	0.55
Geriatric depression scale	2 [3]	1 [1]	0.0003
Social provisions scale	35 [17]	31[11]	0.16

*^a^*P*-values computed using Fisher’s exact test for binary variables and Deuchler–Wilcoxon’s exact test for continuous variables*.

*^b^Computed as [(2 × diastolic blood pressure) + systolic blood pressure]/3*.

A total of 74 (76%) participants reported fatigue that was different than fatigue prior to their MI. The average fatigue score for each subscale was (a) function (Md = 13, IQR = 19); (b) behavioral/severity RPFS (Md = 22, IQR = 25); (c) affective meaning RPFS (Md = 20, IQR = 21); (d) sensory RPFS (Md = 21, IQR = 17); and (e) cognitive/mood RPFS (Md = 18, IQR = 19). Fisher’s exact test indicated no differences in the proportion of women and men reported fatigue after MI (*P* = 0.152). Additionally, women did not have significantly higher levels of fatigue than men (*U* = 2398.5; *P* = 0.85).

A statistically significant unadjusted linear trend was observed for IL-6, mean arterial blood pressure (MAP), GDS, and age (Table 2). Compared with underweight participants, those who were normal, pre-obese, or obese were significantly more likely to report fatigue; however, the effect was not linear. Only depression scores (*P*_trend_ = 0.0004) and mean arterial pressure (*P*_trend_ = 0.015) were found to be linearly independent predictors for fatigue, controlling for age, Il-6 levels, and body mass index.

**Table 2:** **Univariable and multivariable factors contributing to fatigue**.

Characteristics	Univariable RR (95% CI)	Multivariable RR (95% CI)
**Age (years)**		
Q1 (≤70)	1.0 referent	1.0 referent
Q2 (>71–76)	1.1 (0.76–1.5)	1.2 (0.82–1.6)
Q3 (>76–81)	1.1 (0.76–1.5)	1.2 (0.89–1.6)
Q4 (>81)	1.4 (1.1–1.9)^†^	1.2 (0.95–1.6)^¥^
**Body mass index (kg/m^2^)**		
Q1 Underweight	1.0 referent	1.0 referent
Q2 Normal	0.84 (0.71–0.994)	0.88 (0.57–1.3)
Q3 Pre-obese	0.73 (0.60–0.89)	0.83 (0.52–1.3)
Q4 Obese	0.71 (0.57–0.88)^¥^	0.81 (0.51–1.3)^¥^
**Geriatric depression scale**		
Q1 (≤1)	1.0 referent	1.0 referent
Q2 (>1–2)	1.5 (1.1–2.0)	1.5 (1.1–2.0)
Q3 (>2–4)	1.4 (1.01–1.9)	1.5 (1.1–2.1)
Q4 (>4)	1.7 (1.3–2.2)^§^	1.6 (1.2–2.0)^§^
**Interleukin-6 (μg/dL)**		
Q1 (≤3)	1.0 Referent	1.0 Referent
Q2 (>3–5)	1.2 (0.87–1.8)	1.1 (0.81–1.6)
Q3 (>5–7)	1.3 (0.90–1.8)	1.2 (0.89–1.6)
Q4 (>7)	1.4 (1.01–1.9)^†^	1.1 (0.81–1.5)^¥^
**Mean arterial pressure (mmHg)**		
Q1 (≤77)	1.0 referent	1.0 referent
Q2 (>77–85)	0.92 (0.73–1.2)	0.95 (0.72–1.3)
Q3 (>85–94)	0.88 (0.69–1.1)	0.95 (0.71–1.3)
Q4 (>94)	0.56 (0.36–0.87)^‡^	0.62 (0.40–0.96)^†^
**B-natriuretic peptide (pg/mL)**		
Q1 (≤74)	1.0 Referent	–^a^
Q2 (>74–118)	1.1 (0.79–1.5)	
Q3 (>118–251)	1.0 (0.74–1.4)	
Q4 (>251)	1.0 (0.74–1.4)^¥^	
**β-Blockers**		
No	1.0 referent	–^a^
Yes	1.1 (0.75–1.5)	
**Comorbidities score**		
Q1 (≤8)	1.0 referent	–^a^
Q2 (>8–10)	1.1 (0.83–1.5)	
Q3 (>10–13)	0.95 (0.67–1.4)	
Q4 (>13)	1.2 (0.88–1.6)^¥^	
**Epworth sleepiness scale**		
Q1 (≤3)	1.0 referent	–^a^
Q2 (>3–6)	0.99 (0.71–1.4)	
Q3 (>6–9)	1.1 (0.76–1.5)	
Q4 (>9)	1.1 (0.85–1.5)^¥^	
**Heart rate (beats/min)**		
Q1 (≤57)	1.0 referent	–^a^
Q2 (>57–64)	0.90 (0.66–1.2)	
Q3 (>64–70)	1.0 (0.76–1.4)	
Q4 (>70)	0.94 (0.69–1.4)^¥^	
**Hemoglobin (g/dL)**		
Q1 (≤12)	1.0 referent	–^a^
Q2 (>12–13)	0.97 (0.74–1.3)	
Q3 (>13–14)	0.79 (0.56–1.1)	
Q4 (>14)	0.95 (0.71–1.3)^¥^	
**Married**		
No	1.0 referent	–^a^
Yes	1.0 (0.83–1.3)	
**Race**		
Black	1.0 referent	–^a^
White	1.1 (0.84–1.4)	
**Sex**		
Male	1.0 referent	–^a^
Female	0.85 (0.68–1.07)	
**Social provision scale**		
Q1 (≤28)	1.0 referent	–^a^
Q2 (>28–34)	0.94 (0.65–1.4)	
Q3 (>34–43)	1.0 (0.75–1.4)	
Q4 (>43)	1.2 (0.91–1.6)^¥^	

*Likelihood ratio trend test (^¥^*P* > 0.05, ^†^*P* ≤ 0.05, ^‡^*P* ≤ 0.01, and ^§^*P* ≤ 0.001)*.

*CI, confidence interval; RR, relative risk*.

*^a^Not included in multivariable model*.

## Discussion

Results of this study demonstrate that fatigue persists 6–8 months after an MI in the majority (76%) of elderly men and women. These results are considerably higher than the 33% of elderly adults from the community who reported fatigue lasting 1–12 months (21). This is concerning since not only has fatigue been reported to decrease participation in physical activity (21, 30), but this symptom can affect the quality of life of persons post-MI (21) and is associated with poorer cardiovascular outcomes (5). Comparing levels of fatigue with other studies is difficult because of the various measures used in other studies, such as the SF-36 vitality scores (31), a visual analog scale (31), a symptom checklist (32), or a question related to the presence of fatigue (33). At the time of this study, the National Institutes of Health’s Patient-Reported Outcomes Measurement Information System (PROMIS) measure for fatigue was not available, and using this standardized measure may yield comparable results across time and populations. Because fatigue is subjective, identification of only the presence of this symptom may be clinically important when addressing barriers to secondary prevention or symptoms affecting the quality of life.

There was no significant difference in the prevalence of fatigue or the fatigue scores between men and women. While these results are consistent with another study (34), a different study found that women had more fatigue than men; however, they measured vital exhaustion, a concept that includes fatigue or exhaustion but also includes irritability and demoralization (35). Therefore, these differences noted in men and women may or may not have been related to fatigue, but rather to irritability or demoralization. Results from this study indicate that elderly adults, regardless of sex, have fatigue post-MI and should be targeted for interventions.

In multivariable analysis, only depression scores and MAP were significantly different from those persons reporting no fatigue after MI. Post-MI depression is important because those identified as having mild to major depression report a lower adherence to risk reduction behaviors, such as regular exercise (36), and they have higher mortality. While depression is prevalent in older persons with cardiovascular disease and those post-MI, in this study, the proportion of depression is similar to the prevalence of depression in an elderly adult population (37) and less than the prevalence of depression in cardiac populations (38). These differing results may be related to the timing of measurement. Many studies examining depression post-MI measured depression within the first 4 months after MI (39). However, a synthesis of the literature noted that depression does not stabilize until 6 months (9). Therefore, the lower percentage of persons who had scores that indicated depression in this study may be related to measuring depression 6–8 months post-MI. Despite the low percentage of persons with scores screening for depression, this variable was the strongest predictor of fatigue that persists post-MI. Because fatigue may also affect depression, further examination of depression and fatigue is warranted to develop interventions to affect fatigue post-MI.

The other significant variables contributing to fatigue 6–8 months post-MI in the univariable analyses were IL-6 and age. Proinflammatory cytokines increase with age (40), and high levels are associated with sickness behavior: fatigue, fever, and general malaise. Increased levels of cytokines, especially IL-6 and tumor necrosis factor alpha (TNFα), are also associated with frailty (41), decreased muscle endurance (42), and heart failure (43). Although cytokines have been associated with fatigue in those with cancer and diabetes (44, 45), no studies were found that examined IL-6 as an explanatory factor for persistent fatigue after MI. Because increases in inflammatory markers are associated with increased risk of cardiovascular disease (43), expanding the study of cytokines to understand the relationship of cytokines to persistent fatigue after MI is important.

Comorbidities, such as rheumatoid disease (46) and cancer (30), have been associated with fatigue (3), and comorbid conditions increase with age (3). Those with cardiovascular disease, especially elderly adults (37), have a higher number of comorbidities, including diabetes, hypertension, hypercholesterolemia, and obesity. Results of this study, in contrast to some reports in the literature, did not observe an association between comorbidities and fatigue (3, 44). Further studies are needed to facilitate understanding of how comorbid conditions differentially contribute to fatigue post-MI.

Consistent with some (12, 45), but not another study (47), social support was not an important variable in explaining fatigue in the current analysis. The relationship of social support to fatigue is unclear, and continued research is needed to clarify the types of social support, if any, which potentially impact fatigue post-MI. Also, daytime sleepiness, though significant in another study (12), was not significant in this study. Further research is needed to clarify the relationship of sleep and fatigue post-MI.

Cardiac function (BNP and heart rate) was not associated with persistent fatigue in elderly adults post-MI, while MAP was a significant predictor. While fatigue is a common symptom reported in heart failure (10), BNP, an indicator of failure (18), was not associated with fatigue. Heart rate and cardiac contractility are used to compute cardiac output. The independent contribution of contractility and heart rate did not predict fatigue, but the outcome of a decreased cardiac output, decreased MAP, did predict fatigue. Because the MAP is a simple, non-invasive measure and lower MAP is a side effect of post-MI pharmacological therapy, including this measure when examining fatigue post-MI is warranted.

One of the limitations of this preliminary analysis was the sample size of 98, but this is one of the only studies to date comprehensively addressing correlates to fatigue in an aging post-MI population. This study was conducted at a single medical center, which makes it difficult to generalize the study findings to other centers that may not have similar patient profiles. A cross-sectional analysis only measures the variables at one point in time; therefore, patterns of associations can neither be determined nor could we examine differences in fatigue at each month. Using a self-report measure of fatigue may have biased the findings. Because only those persons who agreed to release their names to the research team were contacted, those who did not choose to participate in the research could have reported more or less fatigue. However, those who did not participate did not substantively differ from the participants with respect to age and sex.

## Conclusion

The results of this pilot study provide important information to understand the prevalence of fatigue and factors associated with the persistence of fatigue post-MI. While these results have important implications for practice, this model should be tested in a larger sample. Further studies are needed to better understand the persistence of fatigue after MI over time in elderly adults and to develop interventions targeted at decreasing or eliminating fatigue.

## Author Contributions

PC is the PI of the study, developed the design, collected data, analyzed data, and authored the manuscript. JE is the epidemiologist and biostatistician, conducted the analyses, and assisted with writing the manuscript. WA assisted in analyzing the data and writing the manuscript.

## Conflict of Interest Statement

The authors declare that the research was conducted in the absence of any commercial or financial relationships that could be construed as a potential conflict of interest.
